# The investigation on the hypercoagulability of hepatocellular carcinoma‐related cerebral infarction with thromboelastography

**DOI:** 10.1002/brb3.2961

**Published:** 2023-03-16

**Authors:** Gengyu Cen, Yiting Song, Shijian Chen, Liuyu Liu, Jun Wang, Jian Zhang, Jing Li, Guohui Li, Haihua Li, Hongbin Liang, Zhijian Liang

**Affiliations:** ^1^ Department of Neurology The First Affiliated Hospital of Guangxi Medical University Nanning China; ^2^ Department of Neurology The Second Affiliated Hospital of Guangxi Medical University Nanning China; ^3^ Department of Neurology The Affiliated Tumor Hospital of Guangxi Medical University Nanning China; ^4^ Department of Neurology Wuzhou Red Cross Hospital Wuzhou China; ^5^ Department of Neurology Fusui County People's Hospital Chongzuo China; ^6^ Department of Neurology Cenxi People's Hospital Cenxi China

**Keywords:** cerebral infarction, hepatocellular carcinoma, pathogenesis, thromboelastography

## Abstract

**Aim:**

To investigate the hypercoagulability of hepatocellular carcinoma (HCC)‐related cerebral infarction (HCRCI) with thromboelastography (TEG).

**Methods:**

A multicenter prospective study was conducted in HCRCI patients, HCC patients without cerebral infarction, and acute cerebral infarction (ACI) patients without HCC between January 2016 and December 2019. TEG parameters and laboratory and clinical data were collected and compared among the three groups. To confirm the independent risk factors of HCRCI, multivariate analyses were conducted. Receiver operating characteristic (ROC) curves were utilized to evaluate the area under the curve (AUC) plotted by each independent risk factor.

**Results:**

There were 38 patients recruited in the HCRCI group, and 152 patients were recruited to the HCC group and the ACI group. The levels of plasma neutrophil count, D‐dimer, α‐fetoprotein (AFP), carcinoembryonic antigen, and maximum amplitude (MA)—a parameter of TEG—were significantly higher in the HCRCI group than HCC and ACI groups. Multivariate logistic regression analysis showed that increased neutrophile count, D‐dimer, AFP, and MA were independently associated with HCRCI. ROC curve analysis showed first that AUC of MA for HCRCI was .875, which was larger than the other risk factors, and second that the optimal cutoff value for MA was 61.35, with a sensitivity of 89.50% and specificity of 66.40%.

**Conclusion:**

It was suggested that TEG disclosed that the pathogenesis of HCRIC is exactly related to the hypercoagulability. And with a cutoff value of MA equaling to 61.35, TEG facilitates clinicians to identify HCC patients at high risk of HCRIC.

## Graphical abstract

TEG which can comprehensively and accurately reflect overall blood coagulation status to evaluate coagulation in patients with HCRCI. TEG confirmed that pathology of HCRCI was related to hypercoagulation.

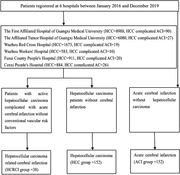

## 1 INTRODUCTION

Cancer‐related cerebral infarction (CI) has received increased attention in the past few years. Now, it is believed that cancer‐related CI may have its own specific clinical features, and most of its pathogenesis is related to hypercoagulability (Selvik et al., 2018, Cutting et al., 2017). Previous studies have found that the mucins secreted by cancer cells into the blood are associated with hypercoagulability in patients with cancer‐related CI (Shao et al., 2011, Jovin et al., 2005, Amico et al., 1989).

Hepatocellular carcinoma (HCC) is a common malignant tumor all over the world (Cong et al., 2016), and the incidence is also rising in China (Fang et al., 2015). It was found that HCC patients were 1.8 times more likely than the general population to have a cerebral infarction within a year after HCC being diagnosed (Zöller et al., 2012), indicating that some patients have HCC‐related cerebral infarction (HCRCI). However, there have been few reports on HCRCI. Does HCRCI also have specific clinical characteristic? Is HCRCI also related to hypercoagulability? This hypercoagulable state is related to elevated serum level of α‐fetoprotein (AFP), which is thought to be one type of mucin secreted by HCC. All of the questions mentioned above are interesting and worth exploring.

Patients with cancer‐related CI are considered to have hypercoagulability because of higher plasma D‐dimer levels (Selvik et al., 2018, Cutting et al., 2017). However, previous studies have shown that routine coagulation tests are insufficient to accurately measure coagulation status in patients with cancer‐related CI (Cheng et al., 2021, Quan et al., 2020, Qin et al., 2018). In order to accurately measure coagulation status in patients with cancer‐related CI, a new and more sensitive method is needed. Thromboelastography (TEG) can be used to measure whole blood coagulation, reflecting comprehensively and accurately overall blood coagulation status (Bowry et al., 2014). TEG has been used successfully to identify hypercoagulable state in patients with prostate cancer (Toukh et al., 2014) and lung‐cancer‐related CI (Quan et al., 2020).

In the present study, we prospectively collected patients with active HCC and with acute cerebral infarction (ACI) without conventional vascular risks as HCRCI group patients to investigate the coagulation status in patients with HCRCI and the underlying pathogenesis of HCRCI with TEG. We hypothesized that the development of HCRCI is related to hypercoagulability caused by elevated plasma AFP, and TEG could more accurately describe the coagulation status of patients with HCRCI compared with routine blood coagulation function tests. This study may contribute to disclosing the pathogenesis of HCRIC exactly relating to hypercoagulability, identifying HCC patients at high risk of HCRIC. And may further contribute to clinicians taking more targeted anticoagulant prophylaxis to prevent the development of HCRCI.

## 2 MATERIALS AND METHODS

### 2.1 Ethical approval

This study was conducted in compliance with the Declaration of Helsinki and approved by the Medical Ethics Committee of the First Affiliated Hospital of Guangxi Medical University. Approval Number: 2021(KY‐E‐177). Written informed consent was obtained from all patients or caregivers.

### 2.2 Patient selection

We recruited patients with HCRCI who were hospitalized between January 2016 and December 2019 in the First Affiliated Hospital of Guangxi Medical University, the Affiliated Tumor Hospital of Guangxi Medical University, Wuzhou Red Cross Hospital, Wuzhou Workers’ Hospital, Fusui County People's Hospital, and Cenxi People's Hospital.

The HCRCI group comprised patients (age: ≥18 years) with active HCC complicated with ACI without conventional vascular risk factors (Guo et al., 2014, Kim et al., 2013, Schwarzbach et al., 2015). The cancer diagnosis of patients always preceded stroke onset. Active HCC was defined as a diagnosis or treatment of HCC during the last 6 months, or known metastatic/recurrent HCC (Guo et al., 2014, Schwarzbach et al., 2015, Lee et al., 2014). Histological testing was selected as the gold standard for HCC diagnosis. The criteria used for the diagnosis of ACI were determined according to the American Heart Association stroke diagnostic criteria (Powers et al., 2018). The etiological classification of CI was ascertained by reference to the Trial of Org 10172 in Acute Stroke Treatment (TOAST) criteria (Adams et al., 1993). The exclusion criteria for the HCRCI group included patients with HCC complicated with CI with conventional vascular risk factors; patients with primary or secondary intracranial tumors; patients with other systemic malignancies; patients with HCC complicated with cerebral hemorrhage and other nervous system diseases; patients with heart, lung, kidney, and other important organ dysfunction; patients were treated with intraarterial/intravenous thrombolysis, mechanical thrombectomy, hemodialysis, infection <14 days, platelet count <50,000/mm^3^, and pregnancy; and patients who refused to be recruited. Patients enrolled in the study strictly followed the double‐blind principle, and were reviewed and confirmed by one neurologist, one oncologist, and one radiologist.

**FIGURE 1:**
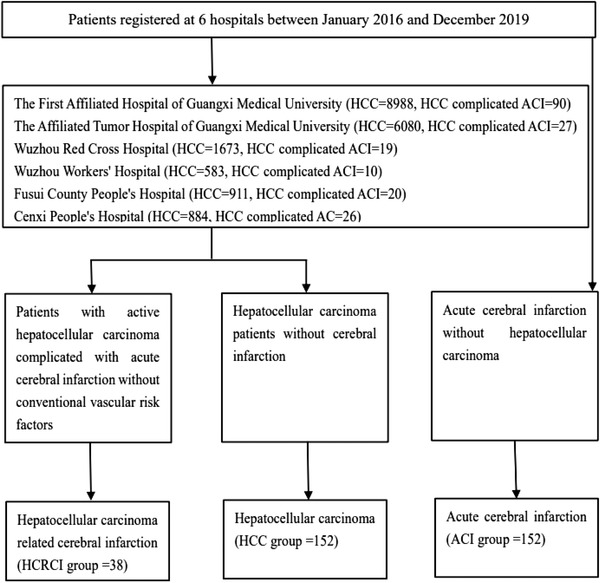
Patient selection. Thirty‐eight patients with hepatocellular carcinoma (HCC)‐related cerebral infarction were recruited, along with 152 with acute cerebral infarction and 152 with HCC as the control group.

We collected HCRCI group and control group according to 1:4. Participants in the ACI group were individually matched to participants in the HCRCI group based on age stratum (≥65 vs. <65 years) and sex, while participants in the HCC group were individually matched to patients in the HCRCI group based on age stratum (≥65 vs. <65 years), sex, and cancer types (Figure 1).

### 2.3 Collection of clinical data

In the HCRCI group and stroke‐only group, blood was collected 72–120 h after the onset of cerebral infarction. In the HCC‐only group, blood was collected immediately after enrollment. Blood samples were immediately tested in laboratory, including routine blood examination (including white blood cell, platelet, and neutrophil counts), blood coagulation examination (prothrombin time [PT], activated partial thromboplastin time [APTT], fibrinogen, international standardized ratio [INR], D‐dimer, and TEG), and concentration of tumor markers (AFP, carcinoembryonic antigen [CEA], cancer antigen 125 [CA125], cancer antigen 153, and cancer antigen 199).

The TEG 5000 analyzer (Haemoscope Corporation, Skokie, IL, USA) and accompanying reagents were used in the present study. Briefly, 1000 μL of whole blood sample of citrate was injected into a kaolin reagent bottle, mixed, and allowed to stand for activation. Then, 340 μL of this solution was injected into the standard specimen cup, and 20 μL of 0.2 mol/L calcium solution was added into the standard specimen cup for immediate detection. After being processed by computer collection and analysis software, TEG parameters were recorded, including coagulation reaction time (*R*‐time), *α*‐angle, time of clot formation (*K*‐time), coagulation index (CI), clot lysis at 30 min (LY30), and maximum amplitude (MA). TEG was performed by the same trained operator. Quality control checks were carried out in accordance with the manufacturer's instructions.

We collected participants’ baseline data, including general data (age, sex, etc.), HCC data (pathological types, whether there were metastasis and treatment), CI data (stroke etiology, infarct patterns, and clinical features), and cranial computed tomography/diffusion‐weighted imaging/magnetic resonance imaging. The National Institute of Health Stroke Scale (NIHSS) was utilized to evaluate the severity of focal neurological dysfunction and the modified Rankin Scale (mRS) was utilized to assess the outcome.

### 2.4 Statistical analysis

Statistical analysis was performed by SPSS 25.0 (IBM, Armonk, NY, USA). Categorical variables were represented by *n* (%); continuous variables were represented by mean ± SD. In the case of continuous data, the significance of differences between groups were evaluated using the independent sample *t*‐test or Mann–Whitney test, or *χ* ^2^ and Fisher's exact tests (two‐tailed) in the case of categorical data. Multivariate logistic regression analysis was utilized to identify independent risk factors for HCRCI. Receiver operating characteristic (ROC) curve was utilized to assess the area under the curve (AUC) plotted for each independent risk factor. The AUC of each independent risk factor was compared using Medcalc version 10.4.8 (Frank Schoonjans). The variable with the largest AUC should be considered to have the best predictive power for HCRCI, and the cutoff value of this variable should be used as a predictor of HCRCI occurrence. All tests were two‐sided. A *p*‐value of <.05 was considered statistically significant. Due to the characteristics of cancer‐related ischemic stroke, no formal sample size calculations were performed.

## 3 RESULTS

We recruited 38 patients with HCRCI (32 males and six females; mean age: 63.05 ± 10.63 years), along with 152 patients with HCC and 152 patients with ACI. There were no statistical differences in age and sex among the three groups (*p* > 0.05).

### 3.1 Comparison of clinical data between HCRCI and ACI groups

The etiology of CI in the HCRCI group was cryptogenic CI according to TOAST classification. Comparison of clinical data between the ACI and HCRCI groups showed no significant differences in neurological dysfunction symptoms (dyskinesia, dysarthria, disturbance of sensation and consciousness, and ataxia) and neurological dysfunction degree (NIHSS score on admission) (*p* > 0.05). According to the mRS score, the rate of good prognosis was lower in the HCRCI group than in the ACI group (9/38 [23.68%] vs. 108/152 [71.05%]; *p* < .05), while the rates of poor prognosis and mortality were higher in the HCRCI group (18/38 [47.37%] vs. 36/152 [23.68%] and 11/38 [28.95%] vs. 8/152 [5.26%]; *p* < .05). D‐dimer level was elevated in the HCRCI group (*p* < .05). For the other laboratory examinations, the differences between the two groups were not significant (*p* > .05) (Table 1).

**TABLE 1:** Clinical data of HCRCI and ACI groups

Item	HCRCI group (*n* = 38)	ACI group (*n* = 152)	*p*
Age (years)	63.05 ± 10.63	62.55 ± 9.96	.785^a^
Sex (male/female)	32/6	128/24	1.000^b^
Cryptogenic ischemic stroke, *n* (%)	38 (100.0)	20 (13.16)	<.001^b^
Clinical feature, *n* (%)			
Dyskinesia	25 (65.79)	103 (67.76)	.816^b^
Dysarthria	12 (31.58)	60 (39.47)	.370^b^
Disturbance of sensation	11 (28.95)	32 (21.05)	.298^b^
Ataxia	11 (28.95)	28 (18.42)	.151^b^
Disturbance of consciousness	8 (21.05)	16 (10.53)	0.140^b^
Swirl, cephalalgia	4 (10.53)	27 (17.76)	.280^b^
Admission NIHSS score	4.10 ± 3.92	4.90 ± 3.28	.198^a^
30‐day mRS score, *n* (%)			
0–2 (good prognosis)	9 (23.68)	108 (71.05)	<.001^b^
3–5 (poor prognosis)	18 (47.37)	36 (23.68)	.004^b^
6 (death)	11 (28.95)	8 (5.26)	<.001^b^
Laboratory examination			
WBC (×10^6^/L)	7.34 (5.61–10.15)	6.48(4.26–8.56)	.235^c^
HGB (g/L)	112.87 ± 21.13	121.59 ± 40.64	.069^a^
Alb (g/L)	33.47 ± 5.68	34.87 ± 9.32	.243^a^
PAB (g/L)	189.52 (72.03–206.37)	213.46 (106.93–372.36)	.126 ^c^
N (×10^9^/L)	5.85 ± 2.32	5.12 ± 2.61	.117^a^
PLT (×10^9^/L)	216.18 ± 67.87	226.42 ± 46.33	.383^a^
PT (s)	13.28 ± 3.32	12.68 ± 1.52	.284^a^
APTT (s)	33.95 ± 4.57	33.08 ± 4.01	.246^a^
Fib (g/L)	3.69 ± 1.17	3.80 ± 0.74	.583^a^
INR	1.04 ± 0.11	0.99 ± 0.23	.055^a^
D‐dimer (ng/L)	1923.78 (1081.35–3335.05)	109.00 (63.75–211.50)	<.001^c^
*R* (min)	4.81 ± 1.01	5.15 ± 1.33	.087^a^
*K* (min)	1.80 (1.43–1.98)	1.70 (1.28–2.00)	.249^c^
*α* (°)	63.20 ± 8.13	63.00 ± 10.41	.899^a^
MA (mm)	64.90 ± 3.12	63.67 ± 7.36	.118^a^
LY30 (mm)	0.30 (0.20–0.60)	0.20 (0.00–0.50)	.128^c^
CI	2.55 ± 1.09	2.41 ± 1.17	.505^a^

Abbreviations: ACI, acute cerebral infarction; Alb, albumin; APTT, activated partial thromboplastin time; CI, coagulation index; Fib, fibrinogen; HCRCI, hepatocellular carcinoma‐related cerebral infarction; HGB, hemoglobin; INR, international normalized ratio; *K*, clot formation time; LY30, clot lysis at 30 min; MA, maximum amplitude; mRS, Modified Rankin Scale; N, neutrophil; NIHSS, National Institute of Health Stroke Scale; PAB, prealbumin; PLT, platelet; PT, prothrombin time; *R*, reaction time; WBC, white blood cells; *α*, clot formation rate.

^a^ Student's *t* test.

^b^ *χ* ^2^ test.

^c^ Mann–Whitney *U* test.

**FIGURE 2:**
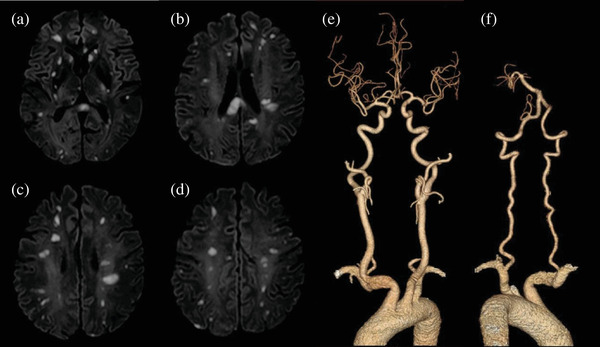
Typical DWI and CTA in a patient with HCRCI. A 51‐year‐old patient without cerebrovascular abnormalities and without vascular risk factors experienced acute cerebral infarction while hospitalized for treatment of hepatocellular carcinoma. DWI, picture A, B, C, and D, shows multiple high‐signal lesions in multiple arterial regions of the brain. CTA, picture E and F, shows bilateral carotid and vertebral artery systems are normal. DWI, diffusion‐weighted imaging; CTA, computed tomography angiography.

Compared with the ACI group, patients in the HCRCI group had a lower rate of single CI in a single arterial area and a higher rate of multiple CIs in multi‐arterial regions. Sometimes the lesions were distributed to the bilateral anterior circulation and posterior circulation (*p* < .05). Small lesions were more common in the HCRCI group (*p* < .05), and the difference was not significant in the size of medium and large lesions and the location of lesions between the two groups (*p* > .05) (Table 2; Figure 2).

**TABLE 2:** Imaging characteristics of HCRCI and ACI groups

Item, *n* (%)	HCRCI group (*n* = 38)	ACI group (*n* = 152)	*p* ^a^
Number of lesions			
Single	6 (15.79)	107 (70.39)	<.001
Scattered	32 (84.21)	45 (29.61)	<.001
Distribution of lesions			
Single vascular region	12 (31.58)	121 (79.61)	<.001
Single	6 (15.79)	105 (69.08)	<.001
Scattered	6 (15.79)	16 (10.53)	.533
Multiple vascular regions	26 (68.42)	31 (20.39)	<.001
Bilateral anterior circulation	5 (13.16)	11 (7.24)	.396
Unilateral anterior and posterior circulation	6 (15.79)	15 (9.87)	.452
Bilateral anterior circulation and posterior circulation	15 (39.47)	5 (3.29)	<.001
Size of lesions			
Small (≤10 mm)	20 (52.63)	35 (23.03)	<.001
Medium (10−30 mm)	12 (31.58)	72 (47.37)	.080
Large (> 30 mm)	6 (15.79)	45 (29.61)	.086
Location of lesions			
Cortex/subcortex	16 (42.11)	47 (30.92)	.190
Deep brain	21 (55.26)	100 (65.79)	.227
Under tentorium cerebelli	15 (39.47)	37 (24.34)	.061
Brainstem	8 (21.05)	23 (15.13)	.377
Cerebellum	7 (18.42)	14 (9.21)	.183

^a^ *χ* ^2^ test was used to compare the two groups.

### 3.2 Comparison of clinical data between HCRCI and HCC groups

The rate of distant metastasis in the HCRCI group was higher than that in the HCC group (*p* < .05). There were no differences between the two groups in the pathological types of HCC and HCC treatment (*p* > .05). Compared with the HCC group, neutrophil count, D‐dimer, AFP, CEA, and MA value of TEG in the HCRCI group were significantly elevated (*p* < .05), while there were no significant differences in other examinations (*p* > .05) (Table 3).

**TABLE 3:** Clinical data of HCRCI and HCC groups

Item	HCRCI group (*n* = 38)	HCC group (*n* = 152)	*p*
Age (year)	63.05 ± 10.63	63.72 ± 9.89	.713^a^
Sex (male/female)	32/6	128/24	1.000^b^
WBC (×10^6^/L)	7.34 (5.61–10.15)	6.74 (4.82–7.91)	.308^c^
HGB (g/L)	112.87 ± 21.13	118.64 ± 43.36	.242 ^a^
Alb (g/L)	33.47 ± 5.68	33.89 ± 4.91	.648^a^
PAB (g/L)	189.52 (72.03–206.37)	186.68 (69.03–197.59)	.852^c^
N (×10^9^/L)	5.85 ± 2.32	3.86 ± 1.19	<.001^a^
PLT (×10^9^/L)	216.18 ± 67.87	195.81 ± 82.75	.162^a^
PT (s)	13.28 ± 3.32	12.62 ± 1.90	.245^a^
INR	1.04 ± 0.11	1.00 ± 0.15	.068^a^
APTT (s)	33.95 ± 4.57	33.63 ± 4.41	.692^a^
Fib (g/L)	3.69 ± 1.17	4.02 ± 1.64	.159^a^
D‐dimer (ng/L)	1923.78 (1081.35–3335.05)	832.00 (527.00–1669.59)	<.001^c^
AFP (ng/mL)	519.12 (115.57–1113.44)	122.71 (74.01–218.37)	<.001^c^
CEA (ng/mL)	2.88 (2.28–4.67)	2.35 (1.96–3.01)	.005^c^
CA125 (U/mL)	14.56 (9.84–24.18)	15.65 (10.55–26.20)	.609^c^
CA153 (U/mL)	14.75 (8.56–26.02)	12.51 (8.76–18.21)	.314^c^
CA199 (U/mL)	32.79 (7.82–59.69)	30.38 (12.80–48.40)	.645^c^
*R* (min)	4.81 ± 1.01	4.93 ± 1.32	.542^a^
*K* (min)	1.80 (1.43–1.98)	1.75 (1.53–2.10)	.524^c^
*α* (°)	63.20 ± 8.13	61.13 ± 9.89	.234^a^
MA (mm)	64.90 ± 3.12	60.16 ± 2.60	<.001^a^
LY30 (mm)	0.30 (0.20–0.60)	0.20 (0.00–1.20)	.243^c^
CI	2.55 ± 1.09	2.36 ± 1.32	.413^a^
Hepatocellular carcinoma, *n* (%)	38 (100)	152 (100)	
Metastasis, *n* (%)	27 (71.05)	59 (38.82)	<.001^b^
Treatment of HCC, *n* (%)			
Surgery alone	9 (23.68)	47 (30.92)	.381^b^
Interventional embolization alone	11 (28.95)	26 (17.11)	.099^b^
Surgery and interventional embolization	10 (26.32)	63 (41.45)	.086^b^
Treatment abandoning	8 (21.05)	16 (10.53)	.140^b^

Abbreviations: AFP, α‐fetoprotein; Alb, albumin; CA125, cancer antigen 125; CA153, cancer antigen 153; CA199, cancer antigen 199; CEA, carcinoembryonic antigen; HCC, hepatocellular carcinoma; HGB, hemoglobin; *K*, clot formation time; N, neutrophil; PAB, prealbumin; *R*, reaction time; WBC, white blood cells; *α*, clot formation rate.

^a^ Student's t test.

^b^ *χ* ^2^ test.

^c^ Mann–Whitney *U* test.

Multiple logistic regression analysis indicated that elevated neutrophils (odds ratio [OR] = 1.502, 95% confidence interval [CI]: 1.003−2.250, *p* = .048), D‐dimer (OR = 1.0005, 95% CI: 1.000−1.001, *p* = .046), AFP (OR = 1.002, 95% CI: 1.001−1.003, *p* = .002), and MA (OR = 1.709, 95% CI: 1.298−2.249, *p* < .001) were independent risks of HCC patients to develop HCRCI (Table 4).

**TABLE 4:** Multiple logistic regression analysis

Factor	*β*	SE	Wald *Z*	*p* value	OR	95% CI
N	.407	0.206	3.902	.048	1.502	1.003–2.250
D‐dimer	.0005	0.0002	3.992	.046	1.0005	1.000–1.001
AFP	.002	0.001	9.754	.002	1.002	1.001–1.003
CEA	.338	0.251	1.809	.179	1.402	0.857–2.294
MA	.536	0.140	14.602	<.000	1.709	1.298–2.249
Constant	−39.206	8.706	20.279	<.000	0.000	

Abbreviations: AFP, α‐fetoprotein; CEA, carcinoembryonic antigen; CI, confidence interval; MA, maximum amplitude; N, neutrophil; OR, odds ratio; SE, standard error.

**FIGURE 3:**
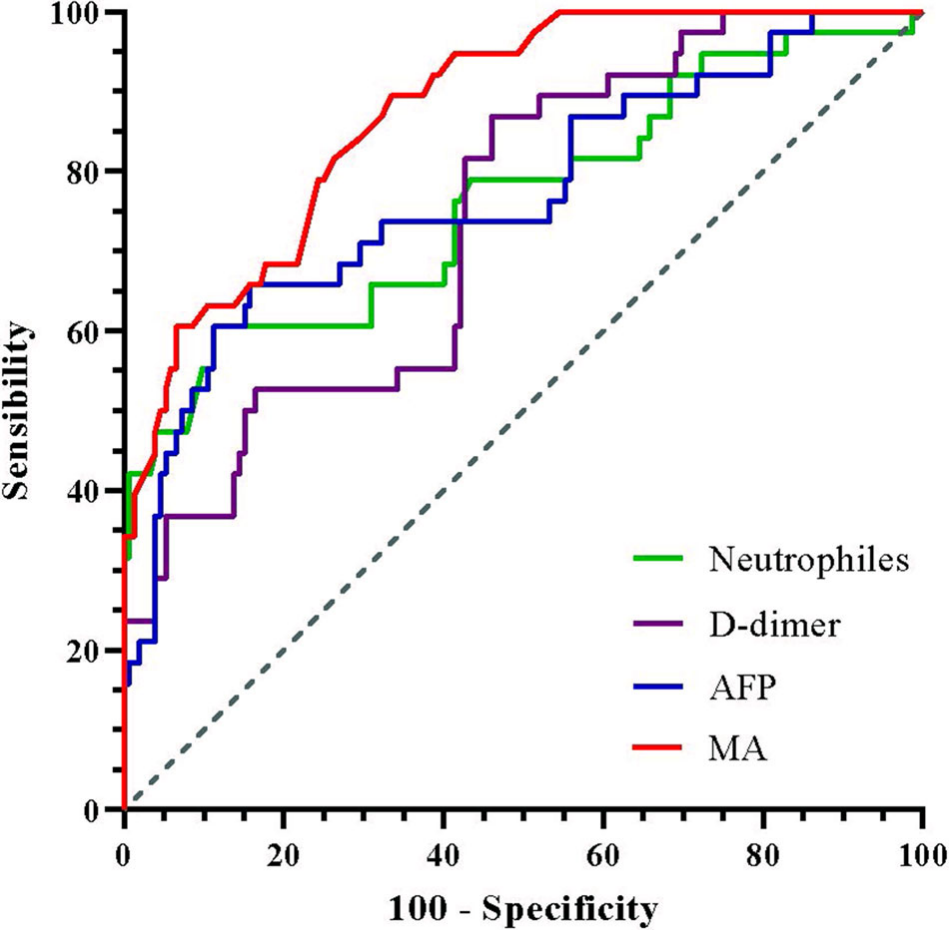
Area under the curve of neutrophils, D‐dimer, α‐fetoprotein (AFP), and maximum amplitude (MA) was .762, .740, .770, and .875, respectively.

Compared to the AUC of neutrophils (.762, 95% CI: .665−.859), D‐dimer (.740, 95% CI: .656−.825), and AFP (.770, 95% CI: .677−.862), the AUC of MA (.875, 95% CI: .819−.932) was the largest, and the optimal cutoff value of MA was 61.35; the sensitivity and specificity of identifying and predicting HCRCI were 89.50% and 66.40%, respectively (Table 5; Figure 3).

**TABLE 5:** Receiver operating characteristic curve analysis of independent correlation factors of hepatocellular carcinoma‐related cerebral infarction

Factor	AUC	95% CI	Sen %	Spe %
N	.762	.665–.859	60.50	88.80
D‐dimer	.740	.656–.825	86.80	53.90
AFP	.770	.677–.862	65.80	84.20
MA	.875	.819–.932	89.50	66.40

Abbreviations: AFP, α‐fetoprotein; AUC, area under the curve; CI, confidence interval; MA, maximum amplitude; N, neutrophil; Sen, sensitivity; Spe, specificity.

## 4 DISCUSSION

Stroke and cancer are the two common leading causes of death or disability around the world. Cancer and CI sometimes share the same risk factors, such as smoking, so sometimes the same patient can suffer from these two diseases at the same time, with cancer complicated by CI (Siriratnam et al., 2019). Moreover, previous studies have shown that cancer can directly or indirectly cause the occurrence of CI (Guo et al., 2014, Kim et al., 2013, Schwarzbach et al., 2015). Furthermore, there will be an increasing number of patients with cancer‐related CI as the treatment of tumors continues to improve. Cancer patients who suffer another ACI will not only suffer additional pain, but also have serious adverse consequences (Kim et al., 2013, Kneihsl et al., 2016). Therefore, cancer‐related CI is receiving increased attention. Previous researches have shown that cancer‐related CI often has clinical features such as increased plasma D‐dimer level and multiple ischemic lesions in multi‐arterial areas, and different cancer cells may have elevation of different tumor markers in serum (Selvik et al., 2018, Schwarzbach et al., 2015, Cocho et al., 2015). In the present study, we found that HCRCI patients had similar characteristics to those with general cancer‐related CI, including increased levels of plasma D‐dimer and multiple lesions in cerebral multi‐arterial regions. Moreover, HCRIC was found to have its unique feature of elevated serum AFP level, which is helpful for clinicians to identify HCRIC from other subtypes of CI.

Besides having specific clinical features, cancer‐related CI also has specific pathogenesis that is worth exploring. Previous studies have found that in addition to the fact that some cancers can directly lead to CI (Budincevic et al., 2016, Zheng et al., 2014), most cancer‐related CI is associated with hypercoagulability based on elevated serum D‐dimer level (Shen et al., 2020, Rosenberg et al., 2020, Gon et al., 2017). However, as has been found in other studies on the pathogenesis of cancer‐related CI, although patients are considered to be hypercoagulable, routine coagulation tests, such as thrombin time, PT, APTT, and INR, are normal in most patients with cancer‐related CI, suggesting that the tests are inadequate to assess blood clotting (Cheng et al., 2021, Quan et al., 2020, Qin et al., 2018). In the current study, increased serum D‐dimer levels suggested that the pathogenesis of HCRCI is related to hypercoagulability. TEG has been shown to have advantages in measuring blood clotting ability by assaying the viscoelastic characteristics of blood clots, providing information on the cumulative impacts of various parameters, including plasma factors and cytokines, at all stages of coagulation and fibrinolysis (Akay et al., 2009).

The MA, one parameter of TEG, is the maximum amplitude of binding of fibrin and platelets to platelet membrane glycoprotein IIb/IIIa receptor, indicating the maximum strength of fibrin/platelet clots. When the MA value increases, it indicates arteriovenous thrombosis and blood hypercoagulability (Zohav et al., 2017). In the present study, TEG was used to detect coagulation in patients with HCRCI, and showed that MA in the HCRCI group was significantly increased, suggesting that patients with HCRCI are in a hypercoagulable state.

However, how hypercoagulability occurs in patients with HCC is uncertain. Studies have found that mucins produced by cancer cells are related to hypercoagulability and raise the risk of CI (Shao et al., 2011, Jovin et al., 2005, Amico et al., 1989). (Jovin et al., 2005) found that the increase of some tumor markers such as CA125 with mucin characteristics was associated with cancer‐related hypercoagulability and contributed to cancer‐related ischemic stroke. (Shao et al., 2011) confirmed through animal studies that mucins secreted by cancer cells can activate neutrophils and platelets and trigger microthrombosis. These studies suggest that mucin‐related hypercoagulability plays a key role in cancer‐associated ischemic stroke. In the current study, we found that AFP was an independent risk factor of HCRCI. AFP is also a similar mucin marker as CA125. Therefore, we speculated that AFP secreted by HCC may contribute to the occurrence of HCRCI by inducing platelets and neutrophils mutual activation.

In addition, neutrophil extracellular traps (NETs) produced by neutrophils are also related to hypercoagulable state and thrombotic diseases, including ischemic stroke (Thålin et al., 2016, Demers & Wagner, 2014). Cancer cells induce formation of NETs, which bind to platelets, activate the coagulation system, and inhibit activation of the anticoagulation system and fibrinolysis, increasing hypercoagulability in patients and leading to thrombosis (Jung et al., 2019). In the present study, neutrophils in HCRCI patients increased, and multivariate logistic regression analysis showed that high neutrophil count can independently increase the risk of HCRCI. This suggests that HCC also induces an increase in neutrophils and the release of NETs and stimulates platelet activation to promote hypercoagulability, and eventually leads to HCRCI.

In the current study, elevated D‐dimer, AFP, neutrophils, and MA value were independently associated with HCRCI, suggesting that these parameters might be utilized as possible biomarkers for HCRCI. To identify high‐risk HCC patients with HCRCI, ROC curve was used to evaluate AUC plotted for each independent correlation factor. The MA value had the largest AUC, with the highest sensitivity and specificity, which proves that MA value can identify patients at high risk of developing HCRCI.

There were some limitations to this study. First, the small patient population limits our ability to generalize the results. Second, MA (determined by TEG) were not different between ACI and HCRCI groups; however, they were different between HCC and HCRCI. The difference in MA(determined by TEG) in HCC and HCRCI remained not excluded be the result thatthe acute stress response to stroke. A larger, prospective, multicenter study will better elucidate the ability of TEG to distinguish HCRCI from other types of CI in terms of etiology and may facilitate prediction of the occurrence of HCRCI.

## 5 CONCLUSION

It was suggested that TEG, on the one hand, disclosed that the pathogenesis of HCRIC is exactly related to the hypercoagulability. And on the other hand, with a cutoff value of MA equaling to 61.35, TEG facilitates clinicians to identify HCC patients at high risk of HCRIC.

## AUTHOR CONTRIBUTIONS

Gengyu Cen and Zhijian Liang conceived and designed the research. Gengyu Cen and Yiting Song collected the data and drafted the initial manuscript. Yiting Song, Shijian Chen, and Liuyu Liu helped to analyze the data. Jun Wang, Jian Zhang, Jing Li, Guohui Li, Haihua Li, and Hongbin Liang helped to collect the data. Zhijian Liang critically revised the manuscript. All authors read and approved the final manuscript.

## CONFLICT OF INTEREST STATEMENT

The authors declare no conflicts of interest.

### PEER REVIEW

The peer review history for this article is available at https://publons.com/publon/10.1002/brb3.2961.

## Data Availability

All data generated or analyzed during this study are included in this article.
